# Structural barriers and facilitators to accessing postsurgical rehabilitation in adults who were treated with surgery for low back pain: protocol for a scoping review

**DOI:** 10.1186/s13643-025-02919-8

**Published:** 2025-08-06

**Authors:** Jessica J. Wong, Jen Rinaldi, Paulo Pereira, Silvano Mior, Lauren Ead, Alanna Veitch, Kent Murnaghan, Pierre Côté

**Affiliations:** 1 School of Physical Therapy, Faculty of Health Sciences, Western University, 1201 Western Road, London, ON N6G 1H1 Canada; 2 Department of Epidemiology and Biostatistics, Schulich School of Medicine and Dentistry, Western University, 1151 Richmond Street, London, ON N6A 5C1 Canada; 3 Institute for Disability and Rehabilitation Research, Faculty of Health Sciences, Ontario Tech University, 2000 Simcoe Street North, Oshawa, ON L1H 7K4 Canada; 4 Populations and Public Health Research Program, ICES Western, 800 Commissioners Road East, London, ON N6A 5W9 Canada; 5 Institute of Health Policy, Management and Evaluation, University of Toronto, 155 College Street, 4th Floor, Toronto, ON M5T 3M6 Canada; 6 Graduate Studies, Canadian Memorial Chiropractic College, 6100 Leslie Street, Toronto, ON M2H 3J1 Canada; 7 Faculty of Social Science and Humanities, Ontario Tech University, 2000 Simcoe Street North, Oshawa, ON L1G 0C5 Canada; 8 Department of Neurosurgery, ULS São João Porto, Alameda Prof. Hernâni Monteiro, Porto, 4200-319 Portugal; 9 Faculty of Medicine, University of Porto, Alameda Prof. Hernâni Monteiro, Porto, 4200-319 Portugal; 10 Department of Gender Studies, Queen’s University, 138 Union Street, Kingston, ON K7L 2P1 Canada; 11 Library Services, Canadian Memorial Chiropractic College, 6100 Leslie Street, Toronto, ON M2H 3J1 Canada

**Keywords:** Post-surgical rehabilitation, Low back pain, Structural barriers, Structural facilitators, Scoping review

## Abstract

**Background:**

Low back pain (LBP) is a major contributor to disability and rehabilitation needs globally. A proportion of patients with LBP undergo surgery and require postsurgical rehabilitation to optimize functioning. However, many encounter barriers to accessing rehabilitation due to structurally generated inequities linked to socioeconomic position. Structural barriers to accessing rehabilitation intersect with diversity-related factors (e.g., gender, ethnicity) to perpetuate stigma and marginalization, leading to tremendous consequences. This literature needs to be reviewed to identify key themes and knowledge gaps focused on structural factors to accessing post-surgical rehabilitation. Our objectives are to conduct a scoping review of the literature to (1) systematically map the literature on the experiences with structural barriers and facilitators to accessing post-surgical rehabilitation of adults who were treated with surgery for LBP (including with or without radiculopathy, symptomatic spinal stenosis); (2) investigate whether these experiences differ when grouped by diversity-related factors (e.g., gender, ethnicity, geographic region).

**Methods:**

We will conduct a scoping review of the literature based on Joanna Briggs Institute (JBI) scoping review guidance and report it according to PRISMA-Scoping Reviews. We will search multiple databases from inception to 2025 for qualitative research exploring experiences with structural barriers or facilitators to rehabilitation access after surgery among adults with LBP. Drawing upon the World Health Organization (WHO) Action on Social Determinants of Health framework, structural factors to accessing rehabilitation will include socioeconomic and political contexts; governance; macroeconomic, social and public policies; and cultural and societal values/norms. Paired reviewers will independently screen articles and extract data. Results will be summarized and grouped by type of LBP and rehabilitation, and by intersections with diversity-related factors (e.g., gender, age, ethnicity, disabilities, geographic region). Our interdisciplinary team will engage with an Advisory Committee of knowledge users with lived experience throughout.

**Discussion:**

Aligned with WHO and EUROSPINE priorities, our scoping review will elucidate the structural barriers and facilitators influencing access to post-surgical rehabilitation for LBP more inclusively. Findings will advance knowledge of structural challenges experienced by adults needing post-surgical rehabilitation, informing rehabilitation and other healthcare strategies to remove barriers and improve functioning globally.

Systematic review registration: Open Science Framework (OSF) https://osf.io/26h9w

**Supplementary Information:**

The online version contains supplementary material available at 10.1186/s13643-025-02919-8.

## Background

Low back pain (LBP) is a major contributor to disability and rehabilitation needs globally [1–3]. A proportion of patients with LBP undergo surgery, particularly for persistent radiculopathy, and surgical rates are increasing [4–12]. In the UK (63 million population), approximately 10,000 lumbar disc excisions were performed in National Health Service hospitals between 2011 and 2012 [4, 5]. The number of surgeries for degenerative lumbar spine disease in England nearly doubled from 1999 (25 per 100,000) to 2013 (49 per 100,000) [6]. Other regions also have increasing surgical rates for LBP, including the USA and Asia [7–12].

Post-surgical rehabilitation is integral to optimizing functioning in patients treated with surgery for LBP. Rehabilitation—defined by the World Health Organization (WHO) as a set of interventions to optimize functioning in persons with health conditions when interacting with their environment—is an important strategy for achieving the United Nations’ Sustainable Development Goal of healthy lives and well-being for all [13, 14]. Post-surgical rehabilitation includes interventions following surgery that improve functioning (e.g., exercises, non-pharmacological or pharmacological therapies) and the environment (e.g., assistive devices, workplace adaptations). Given increasing surgical rates for LBP [6–12], the need for post-surgical rehabilitation will likely continue to grow over time.

However, many patients encounter significant barriers to accessing rehabilitation due to structurally generated inequities [15–19]. Rehabilitation access is determined by structural factors that are primarily non-medical (e.g., occupational and class status, income security, educational opportunities, housing access, social inclusion), which are distributed in ways that produce systemic disadvantage [15–19]. Literature suggests that barriers to accessing rehabilitation are linked to socioeconomic position, health literacy, and finances [17]. Structural barriers intersect with disability to perpetuate stigma and marginalization [18, 20, 21], complicating challenges with participation and recovery following surgery. The physical and psychosocial impact of structural barriers to rehabilitation faced by adults greatly affects care and well-being [17, 18, 20, 21]. A comprehensive view on structural barriers and facilitators to accessing post-surgical rehabilitation for adults treated with surgery for LBP is needed to inform strategies to optimize access to care and functioning. To our knowledge, we are conducting the first review to systematically map the literature in this area.

Our objectives are to conduct a scoping review of the literature to:

1. Systematically map the literature on the experiences with structural barriers and facilitators to accessing post-surgical rehabilitation of adults who were treated with surgery for LBP (including with or without radiculopathy, symptomatic spinal stenosis); and
2. Investigate whether these experiences differ when grouped by social and diversity-related factors (e.g., gender, ethnicity, Indigeneity, place of residence, religion, occupation, education, socioeconomic position, social capital, and class).

## Methods

### Protocol registration and reporting

We will conduct the scoping review according to the updated JBI (formerly Joanna Briggs Institute) scoping review methodology guide [22]. This scoping review will be reported using the Preferred Reporting Items for Systematic Reviews and Meta-analysis Extension for Scoping Reviews (PRISMA-ScR) (adapted to scoping review protocol; Additional file 1) [23, 24]. We pre-registered the protocol with Open Science Framework registries (https://osf.io/26h9w).

### Eligibility criteria

#### Population

We will include adults (aged ≥ 18 years) who were treated with surgery for LBP with or without radiculopathy, including symptomatic spinal stenosis. We define LBP as pain and discomfort below the costal margin and above the inferior gluteal folds, with or without leg pain [25, 26]. Radiculopathy refers to inflammation, injury/dysfunction or compression of the spinal nerve roots that can present as pain, weakness or altered sensation in a myotomal or dermatomal distribution, and may be attributed to spinal stenosis or lumbar disc herniation [27, 28]. We will include adults who received surgery targeting LBP with or without radiculopathy, including surgical procedures for localized LBP, LBP with radiculopathy (e.g., lumbar disc herniation) and symptomatic spinal stenosis; we will exclude deformity surgeries. We will also exclude any major or serious underlying pathology, including traumatic injuries (e.g., fractures or dislocations), infection, tumours or malignancies, osteoporosis, and inflammatory arthritis.

### Concept

We will explore the perspectives of adults who experience structural barriers or facilitators to accessing post-surgical rehabilitation following surgery for LBP with or without radiculopathy. The WHO refers to rehabilitation as a set of interventions aimed to improve functioning in individuals with health conditions when interacting with their environment [13]. We will exclude studies that focus on single interventions only. We will focus on post-surgical rehabilitation in the health care context; to be comprehensive, we will also include other terms such as health care services, treatment, interventions, and community-based programs (e.g., community-based exercise programs) that aim to improve functioning.

### Examples of post-surgical rehabilitation

Post-surgical rehabilitation includes rehabilitation medicine/therapy or programs of care after surgery aiming to (1) improve function through the diagnosis and treatment of health conditions, reducing impairments, preventing or treating complications; and (2) restore and compensate loss of functioning, and prevent or slow deterioration in functioning in every area of a person’s life [13, 14, 29]. Post-surgical rehabilitation can also include assistive technology, which is any item, piece of equipment, or product used to increase, maintain or improve functional abilities [13, 14, 29]. Post-surgical rehabilitation can be provided by different healthcare providers including, but not limited to, general practitioners, orthopaedic surgeons, neurosurgeons, physiotherapists, chiropractors, and occupational therapists. Examples include, but are not limited to, patient education and self-management; compensatory strategies, training and exercises; manual therapies; physical modalities; acupuncture; pharmacological interventions; social support/advice; psychological interventions; environment modifications; provision of resources; workplace adaptation; and assistive technologies [13, 14, 29, 30].

### Examples of structural barriers and facilitators to accessing rehabilitation

Based on the WHO framework for Action on Social Determinants of Health, structural barriers and facilitators include socioeconomic and political contexts, governance, macroeconomic policies, social policies (e.g., related to labour market, housing, land), public policies (e.g., related to education, health, social protection) and cultural and societal values/norms [18]. Additional specific examples include, but are not limited to, socioeconomic position, income security, educational opportunities, housing access, health literacy, and social inclusion. We will focus on structural barriers (factors that hinder) and facilitators (factors that promote) to accessing post-surgical rehabilitation.

### Context

We will investigate whether these experiences with structural barriers and facilitators to accessing rehabilitation intersect with social and diversity-related factors. Informed by the WHO framework for Action on Social Determinants of Health and Cochrane Equity PROGRESS-Plus indicators [18, 31], social and diversity-related factors include gender, ethnicity, Indigeneity, place of residence, religion, occupation, education, socioeconomic position, and social capital/class.

In addition to the stated population, concept, and context, eligible citations must meet the following criteria: (1) English, French, Portuguese, Chinese, or Italian language articles (to increase feasibility of conducting this scoping review); (2) study design: qualitative research study; mixed methods study (where qualitative components can be extracted); systematic or scoping reviews of qualitative studies; or systematic or scoping reviews of mixed methods research (where qualitative components can be extracted); (3) studies with a mixed sample must report separate results for adults with LBP treated with surgery. We will also include books and book chapters, and unpublished literature in the form of theses and dissertations and conference proceedings (full papers). The following will be excluded: guidelines, letters, editorials, commentaries, government reports, meeting abstracts, lectures and addresses, consensus development statements, guideline statements, studies not reporting on methodology; literature reviews, randomized trials; cohort, cross-sectional, and case studies.

### Literature search

With an experienced health sciences librarian, we will search the following databases from database inception to present date: MEDLINE (Ovid), EMBASE (Ovid), CINAHL (EBSCO), PsycINFO (Ovid), and Sociological Abstracts (ProQuest) (Additional file 2). The search strategy will be validated by a second librarian using the Peer Review of Electronic Search Strategies (PRESS) checklist [32, 33]. We will not apply any language restrictions to the search strategy. Reference lists of relevant reviews will be searched to identify additional sources. We will contact authors of primary sources for any additional information as clarification of details in the article if required in any of the review stages. Reference management and citation screening will be conducted using EndNote and EPPI-Reviewer.

### Selection of studies/screening

After removing duplicates, all citations retrieved from the search will be screened based on our eligibility criteria. In phase I, paired reviewers will independently screen titles and abstracts to determine whether citations are “possibly relevant” or “irrelevant”. Paired reviewers will discuss disagreements to reach consensus, with involvement of a third reviewer if necessary. Prior to conducting phase I screening, we will pilot-test 50 randomly selected citations from the literature using two independent reviewers; discrepancies will be discussed until consensus is reached. If the agreement is < 80%, the eligibility criteria will be discussed and modified prior to screening. All citations rated as “possibly relevant” will undergo phase II screening using full texts of articles. Paired reviewers will screen full text articles to determine whether citations are “relevant” or “irrelevant”, and document reasons for exclusion. Discrepancies will be discussed to reach consensus, with involvement of a third reviewer if necessary. Prior to phase II screening, we will conduct pilot-testing similar to phase I screening by randomly selecting 25 full texts and assessing for ≥ 80% agreement.

### Data charting/extraction

We will extract data from relevant studies identified during screening using a predefined data extraction form, which includes sample characteristics and context [e.g., sociodemographics, health status, description of LBP with or without radiculopathy (preoperative pathology, surgical indication) and surgical interventions, type of post-surgical rehabilitation, setting including geographic region], methods (e.g., recruitment, data collection, theoretical framework), and broad findings (e.g., overarching themes on structural barriers or facilitators to accessing rehabilitation, intersectionality with social and diversity-related factors). The extraction form will be pilot tested using five studies by two reviewers, with one reviewer extracting data and one reviewer verifying the extracted data. The research team will review all extracted data to determine whether any modifications to the data extraction form are needed prior to commencing full data extraction. In full data extraction, one reviewer will extract all data from relevant studies, and a second reviewer will verify the extracted data. Any disagreements in data extraction will be discussed to reach consensus, with involvement of a third reviewer if necessary.

### Synthesis

To characterize the existing literature and identify research gaps, we will summarize the extracted data around sample characteristics and context, methods, and broad findings. We will describe these results in relation to the research question and in the context of the overall study purpose. We will group results by type of LBP (e.g., localized LBP versus lumbar disc herniation or symptomatic spinal stenosis), type of surgery (with fusion versus without fusion), and by intersections of accessing different types of post-surgical rehabilitation with social and diversity-related factors (e.g., gender, ethnicity) as they emerge from the data. We will identify gaps in the literature, such as the paucity of data on specific types of LBP conditions, post-surgical rehabilitation following surgeries, or related to certain social and diversity-related factors.

### Advisory committee of knowledge users

Our team includes a diverse Advisory Committee of eight knowledge users, including people with lived experience (adults with LBP) and healthcare providers spanning disciplines of physical medicine and rehabilitation, neurosurgery, physiotherapy, and chiropractic. The Advisory Committee of knowledge users will provide critical input on the methods, interpretation of results, knowledge mobilization, and future research directions in this area. This involves regular virtual meetings to consult with the Advisory Committee on stated areas to ensure that our research is relevant and meaningful to people with LBP and communities.

## Discussion and dissemination of results

Our scoping review will provide a systematic mapping of the structural barriers and facilitators to accessing post-surgical rehabilitation in this population to inform rehabilitation planning and delivery. However, there are limitations and challenges to our proposed scoping review. First, it is possible that we may miss some relevant articles in the literature. However, we will employ a comprehensive search strategy of multiple databases that will be peer-reviewed by a second librarian using the PRESS Checklist [32, 33]. We will also conduct a supplemental search of related reviews to identify any additional relevant studies. Second, only studies in English, French, Portuguese, Chinese, and Italian language due to feasibility will be included, which may exclude relevant studies in other languages. We will include the citation of possibly relevant articles based on title and abstract in the supplementary file for transparency. However, we will first try to find assistance in reviewing studies in other languages through our international network if needed.

Our team will conduct the first scoping review exploring structural barriers and facilitators to accessing post-surgical rehabilitation in adults who received surgery for LBP. Upholding community-based research and equity, diversity, and inclusion principles, we will co-create knowledge with knowledge users (Advisory Committee) as equal partners alongside researchers using integrated knowledge translation. This research is innovative because it seeks to inform and improve existing delivery models of post-surgical rehabilitation and involves emerging applications of an intersectional lens across rehabilitation and social sciences. Given its originality, our project will inform new lines of inquiry on structural barriers and facilitators to accessing post-surgical rehabilitation in adults who received surgery for LBP. We will develop research briefs/infographics to engage knowledge users in our extensive network, including EUROSPINE, WHO Rehabilitation Program, and Cochrane Rehabilitation. The goal is to educate and bring awareness to structural barriers and facilitators to postsurgical rehabilitation and inform potential strategies to improve access to rehabilitation. We also aim to discuss with healthcare professionals and policymakers involved in the delivery and planning of post-surgical rehabilitation for LBP, advancing knowledge on potential challenges patients may experience when accessing care. We will disseminate findings on social media and submit for open access publication in high-impact journals (e.g., European Spine Journal) and conference presentations (e.g., EUROSPINE). Notably, our research will inform strategies and policies to enable equitable access to post-surgical rehabilitation in communities with LBP globally. Our research, in line with the priorities of the WHO and EUROSPINE, will explore structural factors influencing access to post-surgical rehabilitation, with a focus on the intersection of social and diversity-related factors. The findings will enhance our understanding of the structural challenges faced by adults with LBP requiring post-surgical rehabilitation and will help inform strategies to overcome barriers to access worldwide.

## Supplementary Information

Additional file 1: Preferred Reporting Items for Systematic Reviews and Meta-Analyses Extension for Scoping Reviews (PRISMA-ScR) Checklist Adapted to Scoping Review Protocol.

Additional file 2: MEDLINE Search Strategy.

## Data Availability

Not applicable.

## Abbreviations

LBP: Low back pain
PRESS: Peer Review of Electronic Search Strategies
PRISMA-Scoping Review: Preferred Reporting Items for Systematic Review and Meta-analysis Extension for Scoping Review
WHO: World Health Organization
