# Supplementary material for: Structural barriers and facilitators to accessing postsurgical rehabilitation in adults who were treated with surgery for low back pain: protocol for a scoping review
Source: Syst Rev. 2025 Aug 6;14:161. doi: 10.1186/s13643-025-02919-8 (PMC12326590; doi:10.1186/s13643-025-02919-8)
Supplement: Supplementary file 2 — Additional file 2: MEDLINE Search Strategy. [file 13643_2025_2919_MOESM2_ESM.docx]

**Additional File 2. MEDLINE Search Strategy**

Database(s): **Ovid MEDLINE(R) and Epub Ahead of Print, In-Process, In-Data-Review & Other Non-Indexed Citations, Daily and Versions**1946 to July 01, 2024

| 1 | exp Back Pain/su [Surgery] | 3644 |
| --- | --- | --- |
| 2 | exp Back Injuries/su [Surgery] | 9243 |
| 3 | exp Back Muscles/su [Surgery] | 354 |
| 4 | Low Back Pain/su [Surgery] | 2230 |
| 5 | Lumbar Vertebrae/su [Surgery] | 23307 |
| 6 | Lumbosacral Region/su [Surgery] | 1750 |
| 7 | Lumbosacral Plexus/su [Surgery] | 378 |
| 8 | Piriformis Muscle Syndrome/su [Surgery] | 19 |
| 9 | Sciatica/su [Surgery] | 643 |
| 10 | Sacrum/su [Surgery] | 3090 |
| 11 | Coccyx/su [Surgery] | 224 |
| 12 | Sacroiliac Joint/su [Surgery] | 590 |
| 13 | Thoracic Vertebrae/su [Surgery] | 8506 |
| 14 | Intervertebral Disc Displacement/su [Surgery] | 9687 |
| 15 | Intervertebral Disc Degeneration/su [Surgery] | 2733 |
| 16 | Spondylitis/su [Surgery] | 440 |
| 17 | Spondylosis/su [Surgery] | 1623 |
| 18 | Spondylolysis/su [Surgery] | 363 |
| 19 | Spinal Curvatures/su [Surgery] | 577 |
| 20 | Synovial Cyst/su [Surgery] | 848 |
| 21 | Intervertebral Disc/su [Surgery] | 3216 |
| 22 | Zygapophyseal Joint/su [Surgery] | 731 |
| 23 | Radiculopathy/su [Surgery] | 1634 |
| 24 | Polyradiculopathy/su [Surgery] | 262 |
| 25 | Spinal Stenosis/su [Surgery] | 4302 |
| 26 | Spinal Injuries/su [Surgery] | 1909 |
| 27 | Spinal Diseases/su [Surgery] | 6282 |
| 28 | Spine/su [Surgery] | 6734 |
| 29 | 14 or 15 or 16 or 17 or 18 or 19 or 20 or 21 or 22 or 23 or 24 or 25 or 26 or 27 or 28 | 35186 |
| 30 | (lumbar* or (low* adj2 back) or low-back* or (lower* adj2 back) or lower-back* or thoracolumbar* or thoraco-lumbar* or lumbosacral* or lumbo-sacral* or sacral* or sacro-iliac* or sacroiliac*).mp. | 228909 |
| 31 | 29 and 30 | 17770 |
| 32 | (back adj3 (pain* or injur* or ach* or symptom* or myalg* or syndrome* or discomfort* or sore* or impairment* or disorder* or dysfunction* or tear* or imping* or sprain* or strain*)).mp. | 86095 |
| 33 | (backach* or back-ach*).mp. | 4189 |
| 34 | back-pain*.mp. | 78354 |
| 35 | (low-back adj3 (pain* or injur* or ach* or symptom* or myalg* or syndrome* or discomfort* or sore* or impairment* or disorder* or dysfunction* or tear* or imping* or sprain* or strain*)).mp. | 46376 |
| 36 | (lower-back adj3 (pain* or injur* or ach* or myalg* or symptom* or syndrome* or discomfort* or sore* or impairment* or disorder* or dysfunction* or tear* or imping* or sprain* or strain*)).mp. | 5002 |
| 37 | (lumbar* adj3 (pain* or injur* or ach* or myalg* or symptom* or syndrome* or discomfort* or sore* or impairment* or disorder* or dysfunction* or tear* or imping* or sprain* or strain*)).mp. | 10886 |
| 38 | (lumbo* adj3 (pain* or injur* or ach* or myalg* or symptom* or syndrome* or discomfort* or sore* or impairment* or disorder* or dysfunction* or tear* or imping* or sprain* or strain*)).mp. | 2153 |
| 39 | (lumbar* adj3 (disc* adj3 (extru* or degenerat* or displac* or herniat* or prolaps* or sequestered* or slipped* or protru* or avuls*))).mp. | 8100 |
| 40 | (low* adj2 (trunk* adj2 (pain* or facet or (nerve adj2 root*) or osteoarth* or radicul* or stenos* or spondylo* or injur* or discomfort* or dysfunction* or sore* or herniat* or trauma* or sprain* or strain* or ach*))).mp. | 57 |
| 41 | (lower-trunk* adj3 (pain* or injur* or ach* or myalg* or symptom* or syndrome* or discomfort* or sore* or impairment* or disorder* or dysfunction* or tear* or imping* or sprain* or strain*)).mp. | 53 |
| 42 | lumboischialg*.mp. | 98 |
| 43 | (dorsalg* or lumbago*).mp. | 1744 |
| 44 | (coccydyn* or coccygodyn* or coccalg* or coccygalg* or coccygeal*).mp. | 1824 |
| 45 | (coccyx* adj3 (pain* or injur* or ach* or myalg* or symptom* or syndrome* or discomfort* or sore* or impairment* or disorder* or dysfunction* or tear* or imping* or sprain* or strain*)).mp. | 86 |
| 46 | (tailbone* adj3 (pain* or injur* or ach* or myalg* or symptom* or syndrome* or discomfort* or sore* or impairment* or disorder* or dysfunction* or tear* or imping* or sprain* or strain*)).mp. | 20 |
| 47 | (piriformis* adj3 (pain* or injur* or ach* or myalg* or symptom* or syndrome* or discomfort* or sore* or impairment* or disorder* or dysfunction* or tear* or imping* or sprain* or strain*)).mp. | 496 |
| 48 | (sacral* adj3 (pain* or injur* or ach* or myalg* or symptom* or syndrome* or discomfort* or sore* or impairment* or disorder* or dysfunction* or tear* or imping* or sprain* or strain*)).mp. | 1211 |
| 49 | (sacrum* adj3 (pain* or injur* or ach* or myalg* or symptom* or syndrome* or discomfort* or sore* or impairment* or disorder* or dysfunction* or tear* or imping* or sprain* or strain*)).mp. | 140 |
| 50 | (sacro* adj3 (pain* or injur* or ach* or myalg* or symptom* or syndrome* or discomfort* or sore* or impairment* or disorder* or dysfunction* or tear* or imping* or sprain* or strain*)).mp. | 1716 |
| 51 | (SI adj2 (joint adj3 (pain* or injur* or ach* or myalg* or symptom* or syndrome* or discomfort* or sore* or impairment* or disorder* or dysfunction* or tear* or imping* or sprain* or strain*))).mp. | 156 |
| 52 | sciatic*.mp. | 41716 |
| 53 | (thoracic* adj3 (pain* or injur* or ach* or myalg* or symptom* or syndrome* or discomfort* or sore* or impairment* or disorder* or dysfunction* or sprain* or strain*)).mp. | 25261 |
| 54 | ((t-spine* or "t spine") adj3 (pain* or injur* or ach* or myalg* or symptom* or syndrome* or discomfort* or sore* or impairment* or disorder* or dysfunction* or sprain* or strain*)).mp. | 5 |
| 55 | (thoracolumbar* adj3 (pain* or injur* or ache* or myalg* or stenos* or discomfort* or dysfunction* or sore* or impairment* or syndrome* or disorder* or symptom* or tear* or imping* or sprain* or strain*)).mp. | 1228 |
| 56 | (thoraco-lumbar* adj3 (pain* or injur* or ache* or myalg* or stenos* or discomfort* or dysfunction* or sore* or impairment* or syndrome* or disorder* or symptom* or tear* or imping* or sprain* or strain*)).mp. | 70 |
| 57 | (stenos?s* adj2 (lumbar* or lumbo* or sacral* or sacro* or (low* adj2 back) or low-back* or lower-back* or thoracic* or thoracolumb* or L1 or L2 or L3 or L4 or L5)).mp. | 5776 |
| 58 | (neuropath* adj2 (lumbar* or lumbo* or sacral* or sacro* or (low* adj2 back) or low-back* or lower-back* or thoracic* or thoracolumb* or L1 or L2 or L3 or L4 or L5)).mp. | 378 |
| 59 | (radiculopath* adj2 (lumbar* or lumbo* or sacral* or sacro* or (low* adj2 back) or low-back* or lower-back* or thoracic* or thoracolumb* or L1 or L2 or L3 or L4 or L5)).mp. | 1916 |
| 60 | (radiating* adj2 (lumbar* or lumbo* or sacral* or sacro* or (low* adj2 back) or low-back* or lower-back* or thoracic* or thoracolumb* or L1 or L2 or L3 or L4 or L5)).mp. | 338 |
| 61 | (radicular* adj2 (lumbar* or lumbo* or sacral* or sacro* or (low* adj2 back) or low-back* or lower-back* or thoracic* or thoracolumb* or L1 or L2 or L3 or L4 or L5)).mp. | 1102 |
| 62 | (vertebr* adj3 (pain* or injur* or ach* or myalg* or symptom* or syndrome* or discomfort* or sore* or impairment* or disorder* or dysfunction* or tear* or imping* or sprain* or strain*)).mp. | 7113 |
| 63 | (discogen* adj3 (pain* or injur* or ach* or myalg* or symptom* or syndrome* or discomfort* or sore* or impairment* or disorder* or dysfunction* or tear* or imping* or sprain* or strain*)).mp. | 1287 |
| 64 | (curvatur* adj3 (spine* or spinal*)).mp. | 3396 |
| 65 | (synovial* adj3 cyst*).mp. | 2674 |
| 66 | spondyl*.mp. | 57013 |
| 67 | fibrositis*.mp. | 598 |
| 68 | (polyradicul* or poly-radicul*).mp. | 10060 |
| 69 | ((spine* or spinal*) adj4 (condition* or degener* or diseas* or disabl* or disabilit* or disorder* or pain* or injur* or impairment* or instabilit* or symptom* or syndrome* or discomfort* or sore* or ache* or complaint*)).mp. | 146927 |
| 70 | 62 or 63 or 64 or 65 or 66 or 67 or 68 or 69 | 215850 |
| 71 | 30 and 70 | 45603 |
| 72 | (osteoarthrit* adj3 (lumbar* or (low* adj2 back) or low-back* or (lower* adj2 back) or lower-back* or thoracolumbar* or thoraco-lumbar* or lumbosacral* or lumbo-sacral* or sacral* or sacro-iliac* or sacroiliac*)).mp. | 686 |
| 73 | Osteoarthritis, Spine/ | 218 |
| 74 | 32 or 33 or 34 or 35 or 36 or 37 or 38 or 39 or 40 or 41 or 42 or 43 or 44 or 45 or 46 or 47 or 48 or 49 or 50 or 51 or 52 or 53 or 54 or 55 or 56 or 57 or 58 or 59 or 60 or 61 or 72 or 73 | 170929 |
| 75 | 71 or 74 | 195632 |
| 76 | (surger* or surgical* or operat*).ti. | 904902 |
| 77 | su.fs. or Postoperative Period/ | 2347251 |
| 78 | 76 or 77 | 2772105 |
| 79 | 75 and 78 | 48683 |
| 80 | 1 or 2 or 3 or 4 or 5 or 6 or 7 or 8 or 9 or 10 or 11 or 12 or 13 or 31 or 79 | 71383 |
| 81 | (barrier* or facilitator* or participation*).ti,ab,kf. | 648507 |
| 82 | "Social Determinants of Health"/ | 7511 |
| 83 | exp Health Services Accessibility/ | 138308 |
| 84 | (health* adj3 (access or accessibility or equit* or inequit* or dispar* or restrict* or obstacl* or utili?ation*)).ti,ab,kf. | 136487 |
| 85 | (primary care adj3 (access or accessibility or equit* or inequit* or dispar* or restrict* or obstacl* or utili?ation*)).ti,ab,kf. | 3768 |
| 86 | exp "Patient Acceptance of Health Care"/ | 176394 |
| 87 | exp Patient Participation/ | 30109 |
| 88 | Socioeconomic Factors/ | 174418 |
| 89 | socioeconomic disparities in health/ | 246 |
| 90 | exp Health Inequities/ | 42237 |
| 91 | exp Income/ or prejudice/ or disability discrimination/ or social discrimination/ or transportation/ or Health Policy/ or Clinical Governance/ or Governing Board/ or Waiting Lists/ or Rural Population/ or Rural Health Services/ or exp "Costs and Cost Analysis"/ | 532943 |
| 92 | Health Literacy/ | 10143 |
| 93 | exp Health Education/ | 266777 |
| 94 | (income or prejudic* or discriminat* or transport* or policy or policies or governance or board or (wait* adj1 list*) or rural or health services or costs or cost or (social adj2 determinant*) or (race or racial or ethnic* or Indigen* or (residence adj2 place*) or religio* or socioeconomic* or (social adj1 (capital or class)) or lesbian or gay or intersectional* or lgbt* or diversity* or minorit* or racis* or prejudic* or refugee* or african-american* or aboriginal* or inuit or "first nation" or unemploy* or poverty)).).ti,ab,kf. | 2521457 |
| 95 | or/81-94 | 3736070 |
| 96 | exp Rehabilitation/ | 364835 |
| 97 | Rehabilitation Centers/ | 8738 |
| 98 | "Physical and Rehabilitation Medicine"/ | 3671 |
| 99 | (telerehab* or tele-rehab* or (tele adj2 rehab*)).ti,ab,kw. | 2770 |
| 100 | rh.fs. | 210771 |
| 101 | Telerehabilitation/ | 1209 |
| 102 | Rehabilitation Research/ | 285 |
| 103 | Aftercare/ | 13835 |
| 104 | (rehab* or habilitat*).mp. | 395460 |
| 105 | Acetaminophen/ | 21320 |
| 106 | Acupressure/ | 1036 |
| 107 | Acupuncture/ | 2093 |
| 108 | exp Acupuncture Therapy/ | 30472 |
| 109 | Analgesics/ | 55065 |
| 110 | Analgesics, Opioid/ | 63715 |
| 111 | exp Anti-Inflammatory Agents, Non-Steroidal/ | 220650 |
| 112 | Antidepressive Agents/ | 50284 |
| 113 | "Bedding and Linens"/ | 4102 |
| 114 | Behavior Therapy/ | 30972 |
| 115 | exp Biofeedback, Psychology/ | 13386 |
| 116 | Chiropractic/ | 3534 |
| 117 | exp Cognitive Behavioral Therapy/ | 38629 |
| 118 | Combined Modality Therapy/ | 186863 |
| 119 | Community-Based Participatory Research/ | 5912 |
| 120 | Community Participation/ | 18721 |
| 121 | Community Health Services/ | 33420 |
| 122 | Community Participation/ | 18721 |
| 123 | Complementary Therapies/ | 18461 |
| 124 | Cryotherapy/ | 5858 |
| 125 | exp Diathermy/ | 17865 |
| 126 | exp Electric Stimulation Therapy/ | 96308 |
| 127 | Electroacupuncture/ | 5277 |
| 128 | Ergonomics/ | 12957 |
| 129 | exp Exercise/ | 258660 |
| 130 | exp Exercise Therapy/ | 66629 |
| 131 | exp Exercise Movement Techniques/ | 10847 |
| 132 | Fluid Therapy/ | 22200 |
| 133 | High-Energy Shock Waves/tu [Therapeutic Use] | 724 |
| 134 | Hospitals, Rehabilitation/ | 144 |
| 135 | Immobilization/ | 13720 |
| 136 | Hot Temperature/tu [Therapeutic Use] | 3008 |
| 137 | exp Hydrotherapy/ | 21115 |
| 138 | Low-Level Light Therapy/ | 7567 |
| 139 | Magnetic Field Therapy/ | 1330 |
| 140 | Magnetics/tu [Therapeutic Use] | 798 |
| 141 | Massage/ | 6958 |
| 142 | exp Medicine, Chinese Traditional/ | 25166 |
| 143 | exp Musculoskeletal Manipulations/ | 19147 |
| 144 | Muscle Relaxants, Central/ | 8531 |
| 145 | Outpatient Clinics, Hospital/ | 15855 |
| 146 | Patient Education as Topic/ | 88861 |
| 147 | Self Care/ | 36645 |
| 148 | Self-Help Devices/ | 5950 |
| 149 | Physical Therapy Modalities/ | 41747 |
| 150 | Extracorporeal Shockwave Therapy/ | 1059 |
| 151 | Physical Fitness/ | 30318 |
| 152 | Restraint, Physical/ | 12788 |
| 153 | Return to Work/ | 3847 |
| 154 | Transcutaneous Electric Nerve Stimulation/ | 5836 |
| 155 | Vibration/tu [Therapeutic Use] | 1564 |
| 156 | Wheelchairs/ | 5574 |
| 157 | Work Capacity Evaluation/ | 6355 |
| 158 | Work Engagement/ | 1034 |
| 159 | acupressure.mp. | 1931 |
| 160 | "acupunctur*".mp. | 36216 |
| 161 | (advice or advise or advised).mp. | 100595 |
| 162 | alexander technique.mp. | 112 |
| 163 | "assistive device*".mp. | 3767 |
| 164 | "back belt*".mp. | 59 |
| 165 | "back school*".mp. | 318 |
| 166 | (back adj2 work).ab,ti. | 1166 |
| 167 | (braces or brace or bracing).ab,ti. | 10898 |
| 168 | canes.ab,ti. | 690 |
| 169 | chiropr*.ab,ti. | 6830 |
| 170 | "cognitive behavioral therap*".ab,ti. | 13992 |
| 171 | "cognitive behavioural therap*".ab,ti. | 5464 |
| 172 | (cold adj3 (therap* or pack* or compress or massage or immersion or soak or treatment or therap*)).ab,ti. | 6035 |
| 173 | "core stabili*".ab,ti. | 1009 |
| 174 | (corset or corsets).ab,ti. | 675 |
| 175 | crutches.ab,ti. | 1504 |
| 176 | cryotherap*.ab,ti. | 8701 |
| 177 | "deep tissue therap*".ab,ti. | 4 |
| 178 | diathermy.ab,ti. | 3234 |
| 179 | (electric* adj3 (stimulation or EMS or heating pad*)).ab,ti. | 67342 |
| 180 | electro-acupuncture.ab,ti. | 955 |
| 181 | (electroacupuncture or electro-acupuncture).ab,ti. | 7266 |
| 182 | (electrogalvanic stimulation or EGS).ab,ti. | 1306 |
| 183 | (electromagnet* and (radiation or therap*)).ab,ti. | 8723 |
| 184 | electromodalit*.ab,ti. | 2 |
| 185 | electrotherap*.ab,ti. | 1716 |
| 186 | (exercise or exercises or exercising).ab,ti. | 362694 |
| 187 | (flexion-distraction or flexion distraction).ab,ti. | 290 |
| 188 | fluidotherap*.ab,ti. | 35 |
| 189 | galvanic stimulation.ab,ti. | 265 |
| 190 | (H-Wave Device Stimulation or HWDS).ab,ti. | 38 |
| 191 | ((heat* or hot) adj3 (therap* or pack* or compress or massage or lamp or pad or bath or soak or tub or bottle or superficial or therapeutic)).ab,ti. | 5218 |
| 192 | (high energy shock wave* or high-energy shock wave* or HESW).ab,ti. | 159 |
| 193 | "hydrotherap*".ab,ti. | 1191 |
| 194 | (ice adj3 (therap* or pack* or compress or massage or immersion or soak or treatment or therap*)).ab,ti. | 1767 |
| 195 | "interferential current*".ab,ti. | 263 |
| 196 | infrared.ab,ti. | 197761 |
| 197 | iontophoresis.ab,ti. | 4760 |
| 198 | electroanalges*.ab,ti. | 199 |
| 199 | ergonomic*.ab,ti. | 12800 |
| 200 | kinesiotap*.ab,ti. | 217 |
| 201 | ((laser* or lazer*) adj3 (phototherapy or irradiation or biostimulation or light or therap*)).ab,ti. | 37706 |
| 202 | ("low level laser*" or "low level lazer*" or "lumbar support*").ab,ti. | 3687 |
| 203 | (magnetic adj3 (necklace* or therap* or bracelet*)).ab,ti. | 2566 |
| 204 | (manipulat* adj3 (therap* or treatment* or spinal or osteopath*)).ab,ti. | 8736 |
| 205 | "manual therap*".ab,ti. | 3377 |
| 206 | Microcurrent Electrical Neuromuscular Stimulation.ab,ti. | 8 |
| 207 | microwave*.ab,ti. | 49406 |
| 208 | moist air bath.ab,ti. | 0 |
| 209 | moxibustion.ab,ti. | 3850 |
| 210 | ((mobilisation or mobilization) adj4 (osteopath* or orthopedic* or orthopaedic* or lumbar or spinal)).ab,ti. | 415 |
| 211 | ((multimodal* or multi-modal* or multi modal*) adj4 (treatment* or approach or care or therap* or procedure* or package* or manage*)).ab,ti. | 30461 |
| 212 | muscle activation.ab,ti. | 8452 |
| 213 | "muscle energy technique*".ab,ti. | 146 |
| 214 | myofascial release.ab,ti. | 520 |
| 215 | (Neuromuscular Electrical Stimulation or NMES).ab,ti. | 2265 |
| 216 | orthotic*.ab,ti. | 3827 |
| 217 | "passive modalit*".ab,ti. | 48 |
| 218 | (patient* adj3 (educat* or train*)).ab,ti. | 70191 |
| 219 | "Percutaneous Electric* Nerve Stimulation".ab,ti. | 76 |
| 220 | (physical adj therap*).ab,ti. | 30457 |
| 221 | physiotherap*.ab,ti. | 35873 |
| 222 | photo-acupuncture.ab,ti. | 2 |
| 223 | pillow*.ab,ti. | 1538 |
| 224 | pilates.ab,ti. | 892 |
| 225 | (postur* adj3 (correct* or educat* or instruct* or train*)).ab,ti. | 2177 |
| 226 | (pulsed adj3 (electromagnetic or magnetic or radio frequency or energy)).ab,ti. | 3119 |
| 227 | radiant light.ab,ti. | 17 |
| 228 | Russian stimulation.ab,ti. | 5 |
| 229 | (seat* adj cushion*).ab,ti. | 198 |
| 230 | (self-manage* or self manage*).ab,ti. | 29312 |
| 231 | (short wave* or short-wave*).ab,ti. | 6675 |
| 232 | ((shockwave* or shock wave* or shock-wave*) adj3 (ultrasonic or therap* or radiation)).ab,ti. | 3085 |
| 233 | "soft tissue therap*".ab,ti. | 128 |
| 234 | "spray and stretch".ab,ti. | 29 |
| 235 | strain-counterstrain.ab,ti. | 37 |
| 236 | strengthen*.ab,ti. | 136865 |
| 237 | stretching.ab,ti. | 35842 |
| 238 | (tape or taping).ab,ti. | 21236 |
| 239 | thoracolumbosacral orthosis.ab,ti. | 120 |
| 240 | traction.ab,ti. | 24841 |
| 241 | traditional Chinese medicine.ab,ti. | 33907 |
| 242 | (transcutaneous electrical stimulation or TENS).ab,ti. | 21538 |
| 243 | ultrasound.ab,ti. | 324827 |
| 244 | vapocoolant spray.ab,ti. | 67 |
| 245 | "vibration therap*".ab,ti. | 285 |
| 246 | walkers.ab,ti. | 2915 |
| 247 | (walking adj3 aid).ab,ti. | 746 |
| 248 | "warm compress*".ab,ti. | 265 |
| 249 | whirlpool*.ab,ti. | 612 |
| 250 | yoga.ab,ti. | 7180 |
| 251 | (bedding or linen).mp. | 8132 |
| 252 | (behavio* adj2 therap*).mp. | 77893 |
| 253 | (biofeedback* or bibliotherap*).mp. | 12662 |
| 254 | (combined adj2 modalit*).mp. | 190566 |
| 255 | (complementary adj2 therap*).mp. | 23229 |
| 256 | ((light or art or music or recreation*) adj therapy).mp. | 19005 |
| 257 | massag*.mp. | 17932 |
| 258 | self-care.mp. | 53934 |
| 259 | self-help*.mp. | 22255 |
| 260 | wheelchair*.mp. | 10330 |
| 261 | Primary Health Care/ | 94761 |
| 262 | (primary adj2 (healthcare* or care)).ti,ab,kw. | 182200 |
| 263 | (psychologist* or (mental* adj2 therap*) or psychotherap*).mp. | 127332 |
| 264 | (doctor* or physician* or clinician* or practitioner* or (medic* adj1 (provider* or professional*)) or nurse* or nursing or homeopath* or kinesiolog* or (exercise* adj2 provider*) or (massage* adj2 therap*) or (manual* adj2 therap*) or (occupation* adj2 therap*) or (vocation* adj2 therap*) or physiotherap* or (physical* adj2 therap*) or (respiratory* adj2 therap*) or acupunctur* or (personal* adj2 worker*) or (physician* adj2 assistant*) or (medical* adj2 assistant*) or (physiotherapy* adj2 assistant*) or (social* adj2 work*) or (spiritual* adj2 therap*) or (traditional* adj2 heal*) or (health* adj1 (provider* or professional*))).ti,ab,kw. | 1769183 |
| 265 | Clinical Medicine/ | 5528 |
| 266 | Community Medicine/ | 2095 |
| 267 | exp General Practice/ | 79536 |
| 268 | Integrative Medicine/ | 1954 |
| 269 | Osteopathic Medicine/ | 3416 |
| 270 | Telemedicine/ | 40392 |
| 271 | Physicians/ | 105516 |
| 272 | General Practitioners/ | 11332 |
| 273 | Osteopathic Physicians/ | 410 |
| 274 | Physiatrists/ | 121 |
| 275 | Physicians, Family/ | 17481 |
| 276 | Physicians, Primary Care/ | 4534 |
| 277 | Nursing/ | 51973 |
| 278 | Primary Care Nursing/ | 588 |
| 279 | Nursing, Practical/ | 3444 |
| 280 | Nurses/ | 47498 |
| 281 | Physical Therapy Specialty/ | 3034 |
| 282 | Physical Therapists/ | 3521 |
| 283 | Psychology, Medical/ | 328 |
| 284 | Psychotherapy/ | 58781 |
| 285 | Psychotherapists/ | 232 |
| 286 | Social Work/ | 16613 |
| 287 | Social Workers/ | 1204 |
| 288 | Allied Health Personnel/ | 13338 |
| 289 | Physician Assistants/ | 6518 |
| 290 | Physical Therapist Assistants/ | 26 |
| 291 | Medicine, Traditional/ | 12383 |
| 292 | Mind-Body Therapies/ | 1272 |
| 293 | exp "Treatment Adherence and Compliance"/ | 281766 |
| 294 | or/96-292 | 4848548 |
| 295 | exp Affect/ | 37599 |
| 296 | Attitude/ | 54944 |
| 297 | exp Anthropology, Cultural/ | 184263 |
| 298 | Awareness/ | 22390 |
| 299 | Behavioral Research/ | 3556 |
| 300 | Diaries as Topic/ | 202 |
| 301 | Ethnology/ | 1593 |
| 302 | Ethnopsychology/ | 775 |
| 303 | Focus Groups/ | 37682 |
| 304 | Grounded Theory/ | 2933 |
| 305 | Interview, Psychological/ | 15290 |
| 306 | Interview/ | 31114 |
| 307 | Interviews as Topic/ | 68157 |
| 308 | mindfulness/ or motivation/ | 89611 |
| 309 | Narration/ | 10620 |
| 310 | Observation/ | 5921 |
| 311 | Patient Participation/ | 30109 |
| 312 | Patient Preference/ | 11125 |
| 313 | Patient Satisfaction/ | 91241 |
| 314 | Perception/ | 44968 |
| 315 | Personal Narrative/ | 6484 |
| 316 | Personal Narratives as Topic/ | 358 |
| 317 | Patient Satisfaction/ | 91241 |
| 318 | Personal Satisfaction/ | 25433 |
| 319 | exp Psychology/ | 71076 |
| 320 | exp qualitative research/ | 90010 |
| 321 | exp Social Sciences/ | 2682839 |
| 322 | Tape Recording/ | 4379 |
| 323 | Thinking/ | 17959 |
| 324 | Video Recording/ or Videotape Recording/ | 40027 |
| 325 | attitud*.ti. | 68822 |
| 326 | (aware* or belief* or believe* or mindfulness or opinion* or satisf*).ti. | 121387 |
| 327 | ((audio adj record*) or audiorecord* or audiotap*).ab,ti. | 17195 |
| 328 | biographical method*.ab,ti. | 36 |
| 329 | (constant adj2 (comparative or comparison)).ab,ti. | 6591 |
| 330 | experience*.ti. or (lived* adj2 experience*).ti,ab. | 331890 |
| 331 | (perceiv* or perception*).ti. | 143631 |
| 332 | motivation*.ti. | 21257 |
| 333 | (narrat* not (narrative adj review*)).mp. | 68454 |
| 334 | (action* adj2 (note* or research* or study or studies or work*)).mp. | 15812 |
| 335 | perspectiv*.ti. | 179113 |
| 336 | (opinion* adj3 patient*).mp. | 4884 |
| 337 | (patient* adj3 preferenc*).mp. | 31117 |
| 338 | qualitativ*.mp. | 432380 |
| 339 | (semi-structur* or semistructur* or unstructur*).mp. | 116871 |
| 340 | ((content* or conversation* or discourse* or semantic*) adj3 analys*).mp. | 68923 |
| 341 | (theme* or thematic*).mp. | 198867 |
| 342 | ethno*.mp. | 212832 |
| 343 | ((behavioral* or behavioural* or descriptive*) adj3 (note* or research* or study or studies or work* or analy*)).mp. | 188223 |
| 344 | ((content* or discourse* or semantic*) adj3 (note* or research* or study or studies or work* or analy*)).mp. | 94415 |
| 345 | ((field* adj3 (note* or research* or study or studies or work* or analy*)) or fieldwork*).mp. | 136938 |
| 346 | (focus adj group*).mp. | 75632 |
| 347 | (framework* adj analy*).mp. | 3768 |
| 348 | (grounded* adj theor*).mp. | 16541 |
| 349 | interview*.mp. | 521367 |
| 350 | (key adj informant*).mp. | 12058 |
| 351 | (open adj end*).mp. | 23695 |
| 352 | (purposive* adj sampl*).mp. | 16480 |
| 353 | (theoretical adj2 (sampl* or saturation)).mp. | 1610 |
| 354 | (patient* adj3 (expectation* or journey*)).mp. | 13694 |
| 355 | (mix* adj3 (method* or research* or study or studies or work* or analy*)).mp. | 103444 |
| 356 | mixed-method*.mp. | 49276 |
| 357 | ((participatory* or implementation* or naturalistic or feminist*) adj3 (research* or study or studies or work* or analy* or inquiry)).mp. | 46649 |
| 358 | (observational adj3 analy*).mp. | 14927 |
| 359 | (observation adj3 (participant* or non-participant*)).mp. | 5001 |
| 360 | (case study or case studies or case file review or case management).mp. | 167603 |
| 361 | (process adj2 evaluat*).mp. | 16178 |
| 362 | (metasynthes* or meta-synthes*).mp. | 2381 |
| 363 | (document* adj2 collect*).mp. | 1589 |
| 364 | (open-form or (open* adj2 form) or open-text* or (open* adj2 text*)).mp. | 3119 |
| 365 | ((open adj end*) or open-end*).mp. | 23695 |
| 366 | interview.pt. | 31114 |
| 367 | (multimethod* or multi-method*).mp. | 5781 |
| 368 | or/295-367 | 4778363 |
| 369 | 80 and 294 and 368 | 1753 |
| 370 | 80 and 95 and 368 | 845 |
| 371 | 369 or 370 | 2238 |
| 372 | (surgery* or surgeries* or surgical* or post-surg* or postsurg* or arthrodes* or (fusion* adj2 (lumb* or spine or spinal* or back)) or (endoscop* adj2 (lumb* or spine or spinal* or back)) or fixation* or screw* or dis?ectom* or dis?otom* or electroanalges* or (failed adj back) or foraminectom* or foraminotom* or microdis?ectom* or microendoscop* or nucleotom* or perioperat* or peri-operat* or postoperat* or post-operat* or (post adj2 operat*) or laminectom* or laminoplast* or laminotom* or postlaminectom* or arthroplast* or vertebroplast* or resection* or re-section* or reconstruction* or decompress* or repair* or augmentation* or instrumentation* or transection* or thoracotom* or traction*).ti. | 1375652 |
| 373 | (lumbar* or (low* adj2 back) or low-back* or (lower* adj2 back) or lower-back* or thoracolumbar* or thoraco-lumbar* or lumbosacral* or lumbo-sacral* or sacral* or sacro-iliac* or sacroiliac*).ti. | 89905 |
| 374 | 372 and 373 | 23565 |
| 375 | 374 and 368 and 95 | 313 |
| 376 | 374 and 368 and 294 | 651 |
| 377 | 375 or 376 | 832 |
| 378 | 371 or 377 | 2383 |
| 379 | (comment or editorial or letter or guideline or practice guideline or case reports).pt. | 4481764 |
| 380 | 378 not 379 | 2265 |
| 381 | exp Animals/ | 27309522 |
| 382 | exp Humans/ | 22072802 |
| 383 | 381 not 382 | 5236720 |
| 384 | 380 not 383 | 2252 |
